# PROTECTING THE HEALTH OF VIETNAMESE REFUGEES BY LOCAL HEALTH PROFESSIONALS AND UNIVERSITY STUDENTS

**DOI:** 10.1093/geroni/igae098.0778

**Published:** 2024-12-31

**Authors:** Christina Miyawaki, Kim Nguyen, Tuong-Vi Ho, Angela McClellan

**Affiliations:** University of Houston, Houston, Texas, United States; University of Houston, Houston, Texas, United States; Texas Woman’s University, Houston, Texas, United States; University of Houston, Houston, Texas, United States

## Abstract

Nearly 50 years after the fall of Saigon (1975), studies continue to show the high prevalence of physical (ADL, IADL), mental (depressive symptoms), and cognitive (dementia) disabilities in older Vietnamese refugees. Adverse lifelong experiences in Vietnam and host countries may have impacted these results. Some refugees who arrived during their youth became health professionals while their children attend universities, predominantly enrolled in pre-health courses. Leveraging their bilingual/bicultural and health background, they have hosted free health fairs for their monolingual older Vietnamese for the past 16 years in Houston, Texas, the 2nd largest Vietnamese-populated city in the nation. Despite the high prevalence of dementia, no free cognitive healthcare has been provided. The purpose of the study is to report the collaborative efforts to fill the gap using the Cultural Exchange Model (CEM) and improve dementia literacy. The CEM teaches that to create knowledge, two constituencies need to merge within the Community. One arm of Vietnamese stakeholders (older adults, medical doctors) already exists. We formed another arm, Cognitive Health Initiative (CHAIN): 28 bilingual/bicultural Vietnamese university students, who were trained in Montreal Cognitive Assessment (MoCA). During 2023, we jointly attended 11 health fairs with Vietnamese medical professionals and offered 337 MoCA in Vietnamese and English. The results showed that older Vietnamese (≥65) scored the lowest (22.4/30) among participants (White=24.8; Black=23.9; Hispanic=23.3). Utilizing existing, underlying resilience and the unique affinity among local Vietnamese health professionals, our next steps include continuing to offer a “Memory Booth” by local Vietnamese pre-health students, thereby strengthening intergenerational relationships.

